# Antifungal efficacy of a novel type of nanosalt against human fungal pathogens and its antifungal mechanisms

**DOI:** 10.1007/s00253-026-13924-9

**Published:** 2026-06-24

**Authors:** Qiang Tang, Zhuo Dai, Xin Pan, Li Wang, Jiawei Wang, Mengjuan Fu, Zhanao Wu, Ling Lu

**Affiliations:** 1 Jiangsu Key Laboratory for Pathogens and Ecosystems, College of Life Sciences, Nanjing Normal University, Nanjing, China; 2 Nanjing Stomatological Hospital, Affiliated Hospital of Medical School, Institute of Stomatology, Nanjing University, Nanjing, China; 3 Department of Stomatology, Zhenjiang 359 Hospital, Zhenjiang, China

**Keywords:** Nanosalt, *Aspergillus fumigatus*, Human fungal pathogens, Antifungal activities, Disinfectant

## Abstract

**Abstract:**

The incidence of invasive fungal infections is rising steadily, driven by the expanding immunocompromised patient population and the limited availability of effective antifungal interventions. Among medically important fungi, airborne *Aspergillus* conidia are especially relevant to environmental quality control because they can disseminate through air and contaminate healthcare settings. Here, we designed and synthesized a novel ultrafine sodium chloride powder (nanosalt) by anti-solvent precipitation coupled with high-energy nanonization and evaluated its in vitro antifungal activity in a controlled spray co-exposure system using *Aspergillus* spp. as the primary airborne fungal species. In addition, *Candida albicans* and *Cryptococcus neoformans* were also to be tested for the antifungal broad-spectrum by this system even though they do not belong to typical hospital airborne transmission. Nanosalt showed marked fungicidal activity against the tested human fungal pathogens. Further morphological observations in nanosalt-treated *A. fumigatus* conidia indicated severe cell wall and membrane damage, pore-like structural disruption, leakage-associated loss of integrity accompanied with a time-dependent increase of fungal intracellular reactive oxygen species. Findings in this study mainly identify nanosalt not only might be efficient anti-airborne fungal pathogens but also could be a potential broad-spectrum antifungal reagents. Although currently used aerosol chamber model still has limitation compared actual clinical environment, future practical air-disinfection applications in clinics and related biosafety evaluations are still required.

**Key points:**

• *Novel nanosalt particles were generated by anti-solvent precipitation coupled with high-energy nanonization.*

• *Nanosalts exhibit broad fungicidal activity by disrupting fungal cell walls and membranes.*

• *Nanosalt is a candidate for dry environmental antifungal disinfectant.*

## Introduction

Over recent decades, the global incidence of invasive fungal infections has steadily increased, a concerning trend largely attributed to the growing population of immunocompromised individuals, including those patients undergoing chemotherapy, organ transplantation, or living with HIV/AIDS (Fang et al. 2023; Spallone and Schwartz 2021). This epidemiological shift poses a serious public-health challenge due to the limited number of effective antifungal drugs, difficulties in timely access to therapy, drug-related toxicities, and the increasing emergence of antifungal resistance in key pathogens (Pathakumari et al. 2020; Sedik et al. 2024).

Among fungal pathogens of major clinical concern, *Candida*, *Cryptococcus*, and *Aspergillus* represent a critical priority group among the World Health Organization (WHO) fungal priority pathogens list (World Health Organization 2022). *Candida albicans* is a major opportunistic pathogen that can persist as a commensal organism within the host and transition to pathogenic growth under conditions of immune dysregulation or microbial imbalance. Its infections are therefore more commonly associated with endogenous overgrowth, bloodstream invasion, and contact-related transmission rather than airborne dissemination (Lee et al. 2021; Lopes and Lionakis 2022). By contrast, *Cryptococcus neoformans* is typically acquired through inhalation of environmental propagules and can subsequently disseminate from the lung to the central nervous system, where crossing of the blood–brain barrier may lead to life-threatening cryptococcal meningitis (Iyer et al. 2021; Zhao et al. 2023). *Aspergillus* spp., particularly *Aspergillus fumigatus*, *Aspergillus flavus* and other clinically relevant species in this genus, are especially relevant to airborne environmental control because they produce abundant conidia that are readily aerosolized, environmentally persistent, and capable of contaminating indoor healthcare environments (Wangsanut and Pongpom 2024). The increasing emergence of azole-resistant and multidrug-resistant *Aspergillus* isolates further underscores the need for established preventive strategies that act before infection (Lockhart et al. 2023). Thus, in this study, conidia of *Aspergillus* spp. were selected as the primary airborne-relevant model for evaluating nanosalt-mediated antifungal efficacy under controlled co-exposure conditions, whereas *C. albicans* and *C. neoformans* were included to determine whether nanosalt also exhibits broader in vitro antifungal activity against other high-priority human fungal pathogens.

Compared to many conventional disinfectants for aerosolized application which may have respiratory irritation, instability, and safety (Fait et al. 2019; Silva-Beltrán et al. 2023), nanomaterials offer new opportunities for antimicrobial control through size-dependent surface interactions; however, metal-based nanoparticles with antifungal potential remain constrained by synthesis complexity, biocompatibility concerns, and transformational uncertainty (Huang et al. 2023; Schaefer et al. 2024; Siddiqi et al. 2018). Sodium chloride is attractive in this regard because it is inexpensive, readily available, and highly biocompatible. Moreover, hypertonic sodium chloride has long been known to exert antimicrobial effects largely through osmotic stress-associated dehydration and cellular injury (Alfawaz et al. 2024; Li et al. 2019; Rodrigues et al. 2017; Silva et al. 2020). Converting sodium chloride into a stable, aerosolizable dry nanoscale material could therefore provide a simple and potentially safer platform for environmental fungal contamination control.

Based on this rationale, we synthesized ultrafine sodium chloride particles by anti-solvent precipitation combined with high-energy nanonization. Our working hypothesis was that aerosolized nanosalt could reduce the viability of airborne-relevant *Aspergillus* conidia in a simplified co-exposure chamber while retaining measurable fungicidal activity against selected additional medically important fungi. Most importantly, findings in this study demonstrated that this novel type of nanosalt displayed the highly antifungal efficacy against *Aspergillus* spp. which belonged to the most common airborne fungal pathogens in the environment, suggesting nanosalt could be used as the *Aspergillus*-depleted air-disinfection reagents.

## Materials and methods

### Preparation of nanosalt particles

Nanosalt particles were synthesized using anti-solvent precipitation coupled with high-energy nanonization technology (Liu et al. 2022). Briefly, a saturated NaCl solution was prepared in water at room temperature, followed by addition of alcohol as the anti-solvent together with a modifier under continuous stirring. The precipitated crystals were collected by rapid filtration, dried in a vacuum oven at 80 °C for 15 min, and then refined using a grinding instrument to obtain nanoscale sodium chloride powder. Dynamic particle monitoring showed that the final preparation mainly comprised particles in the 400–1000 nm range, which was the aerosolized material used throughout the study.

### Strains and culture conditions

The fungal strains used in this study included *Aspergillus fumigatus* A1161 as the primary reference strain (Song et al. 2016), one azole-susceptible environmental *A. fumigatus* isolate D2-2S and one azole-resistant environmental *A. fumigatus* isolate ED-29D (Chen et al. 2024), and representative laboratory strains of *Aspergillus flavus* ATCC 204304, *Aspergillus niger* FGSC A1143, *Candida albicans* SC5314, and *Cryptococcus neoformans* H99. All strain information is listed in Table 1 and maintained in the laboratory collection of the Jiangsu Key Laboratory for Pathogens and Ecosystems, College of Life Sciences, Nanjing Normal University, China, and are available from the corresponding authors upon reasonable request for academic reproducibility. Taxonomic affiliation followed the source records and routine laboratory identification of the collection.

**Table 1:** Strains used in this study

Strain name	Description	Source/reference
*Aspergillus fumigatus* A1161	***Δku80, A1160::pyrG***	**(** Song et al. 2016**)**
*Aspergillus fumigatus* D2-2S	**Environmental azole-susceptible isolate**	**(** Chen et al. 2024**)**
*Aspergillus fumigatus* ED-29D	**Environmental azole-resistant isolate**	**(** Chen et al. 2024**)**
*Aspergillus flavus* ATCC 204304	**Reference strain**	**American Type Culture Collection (ATCC), USA**
*Aspergillus niger* FGSC A1143	**Reference strain**	**Fungal Genetics Stock Center (FGSC), USA**
*Candida albicans* SC5314	**Reference strain**	**Fungal Genetics Stock Center (FGSC), USA**
*Cryptococcus neoformans* H99	**Reference strain**	**Fungal Genetics Stock Center (FGSC), USA**

*Aspergillus* strains were routinely cultured on solid YAG medium (2% glucose, 0.5% yeast extract, trace elements, and 2% agar) at 37 °C until abundant conidia formed. For conidial collection, plates were gently flooded with sterile 0.1% Tween-80, the colony surface was gently rinsed to release conidia, and the suspension was filtered to remove hyphal fragments. Conidia were then counted with a hemocytometer and adjusted to the indicated inoculum densities in sterile buffer before nebulization or downstream staining assays. *C. albicans* and *C. neoformans* were cultured on SDA medium (4% glucose, 1% peptone, and 2% agar) at 37 °C; yeast cells were harvested by washing with phosphate-buffered saline (PBS), counted, and adjusted to the indicated cell concentrations before nebulization.

### Antifungal activity assessment

Antifungal activity was evaluated in a quantitative spray co-exposure apparatus using a sealed 370 cm^3^ reaction chamber. Nanosalt dry powder was dispersed using an electric powder sprayer, while fungal suspensions were nebulized simultaneously with a medical nebulizer for 5 s. After spraying, the chamber was kept static to allow contact between fungal cells and nanosalt particles; fungicidal activity was quantified after static treatment for 2 h. The fungal cells were then collected, washed with PBS, plated onto the appropriate solid media, incubated at 37 °C, and colony-forming units (CFUs) were counted to calculate survival rate. Because nanosalt was administered as aerosolized dry powder rather than as a dissolved liquid, the exposure dose was expressed as particle concentration in the chamber instead of mg/mL. The consistent exposure conditions used throughout the antifungal assays consisted of a 5-s nanosalt spray in a sealed 370 cm^3^ chamber, followed by 2 h of static treatment before CFU quantification.

### Intracellular reactive oxygen species (ROS) assay

The reaction solutions containing nanosalts and *A. fumigatus* conidia at different mixing time intervals were collected and centrifuged at 12,000 rpm for 2 min. The supernatant was discarded, and intracellular ROS levels were measured using a ROS assay kit (S0033, Beyotime, China). DCFH-DA probe (10 μM) was added, and samples were incubated at 37 °C for 50 min with gentle inversion every 10 min for sufficient probe-cell contact. Subsequently, the cells were washed three times with 1 × PBS to thoroughly remove any probe that had not entered the cells. Finally, the cells were resuspended in 1 mL of PBS for detection. Fluorescence was measured using a BD FACSVerse flow cytometer (BD FACSVerse, USA) with an excitation wavelength of 488 nm and an emission wavelength of 525 nm.

### Scanning electron microscopy (SEM)

Freshly harvested *A. fumigatus* conidia were evaluated in a quantitative spray co-exposure apparatus. Following exposure, both untreated and nanosalt-treated conidia were collected and filtered, followed by resuspension in 0.1% Tween-80 solution. The suspension was centrifuged at 12,000 rpm for 2 min, and the supernatant was discarded. The pelleted conidia were then resuspended and fixed in 1 mL of 3% glutaraldehyde. Morphological changes of conidia from different treatment groups were subsequently observed using a Hitachi Regulus-8100 scanning electron microscope (Hitachi, Tokyo, Japan).

### Calcofluor white (CFW) staining

Conidia were collected after being evaluated in a quantitative spray co-exposure apparatus with nanosalt particles, and then were transferred onto a coverslip. A 150 μL aliquot of CFW staining solution (10 μg/mL) was added onto the coverslip surface and incubated at 37 °C for 5 min. The CFW stain was discarded, and the coverslip was washed twice with 1 mL of PBS. The samples were fixed with 4% paraformaldehyde for 30 min. The coverslip was inverted onto a glass slide and sealed with nail polish. Observation was performed under a fluorescence microscope (Zeiss Jena, Germany) at settings of 365 nm excitation and 460 nm emission.

### Propidium iodide (PI) staining

Related conidia were collected in a similar manner as described for CFW staining. The PI staining solution (25 μg/mL) was added, and the samples were incubated with the dye at 37 °C for 15 min. After incubation, the staining solution was removed, and the coverslips were washed twice with 1 × PBS buffer. All samples were subsequently fixed with 4% paraformaldehyde for 30 min, mounted onto glass slides, and sealed. Stained samples were visualized using a fluorescence microscope (Zeiss Jena, Germany) configured for 535 nm excitation and 605 nm emission.

### Statistical analysis

All statistical analyses were performed using GraphPad Prism software (GraphPad Software, San Diego, CA, USA). Data are presented as mean ± standard deviation from three independent experiments unless otherwise indicated, and statistical significance was defined as a P < 0.05. For comparisons between two groups, an unpaired two-tailed t-test was used. For experiments involving more than two groups or multiple inoculum/time conditions, one-way ANOVA followed by an appropriate multiple-comparison post hoc test was used.

## Results

### Preparation and characterization of nanosalt

**Fig. 1:**
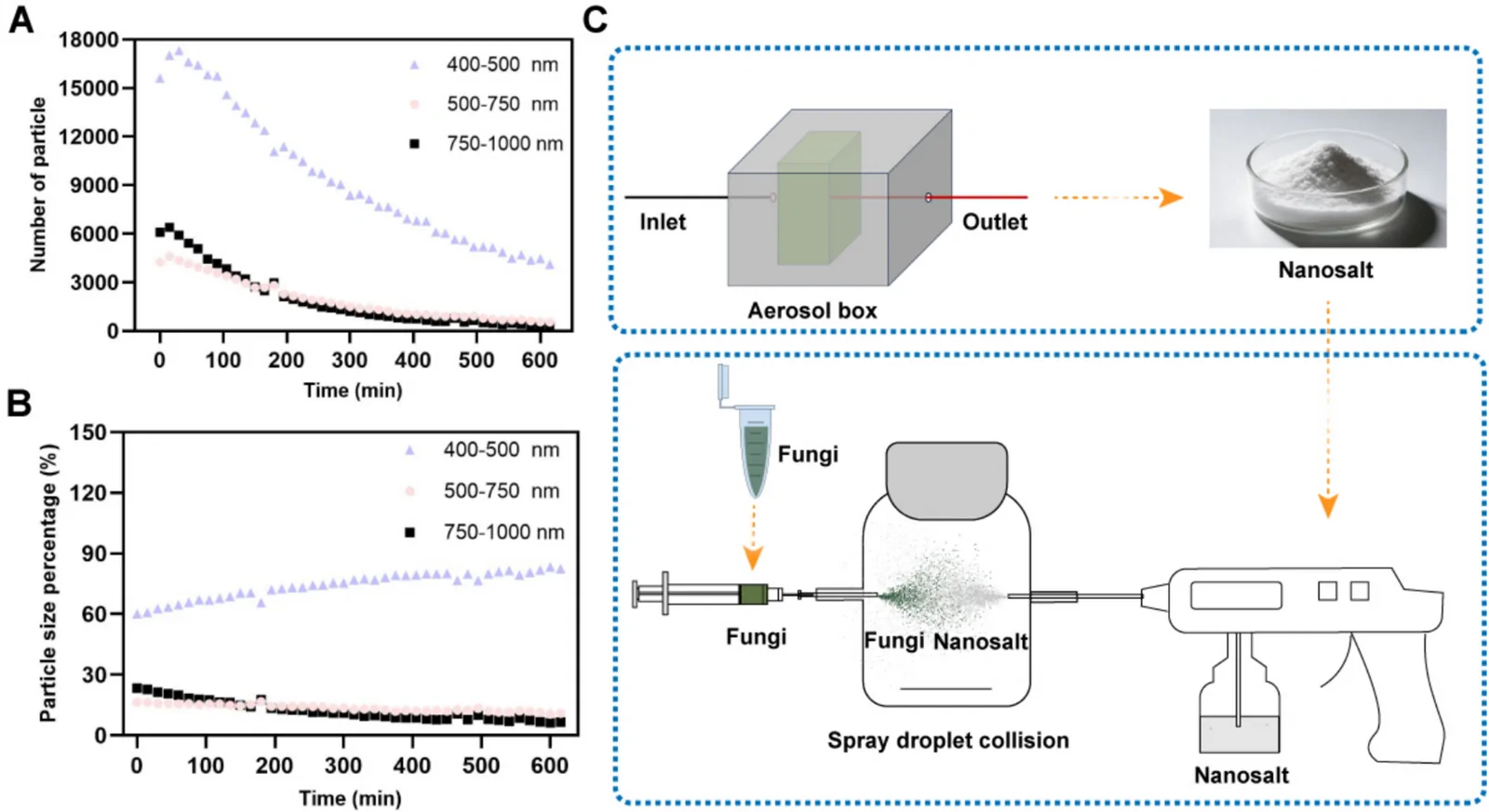
Dynamic particle size distribution during nanosalt preparation and schematic of atomized nanosalt-fungus interaction. **A** Temporal distribution of particles across different size ranges. **B** Particle size proportion of nanosalts with various sizes. **C** Schematic of the atomized nanosalt-fungus-collision assay

Nanoscale sodium chloride was synthesized via anti-solvent precipitation coupled with high-energy nanonization, as described in the methods section. Dynamic particle distribution monitoring was conducted to characterize its physical properties, obtaining the settling velocity and acceleration of various particle sizes (Table 2). As shown in Fig. 1A, the prepared nanosalt particles consisted of a mixed particle size population mainly within the 400–1000 nm range. Among these particles, the 400–500 nm suspended nanosalt particles were the predominant subpopulation, reaching a peak at 17,310, accounting for 62.7% of the total after 30 min of dispersion. These particles maintained a proportion between 60.0–82.5% throughout the observation (Fig. 1B), revealing a stable major subpopulation, while other bigger particles kept a low percentage. After preparation of aforementioned nanosalt particles as shown in the upper panel of Fig. 1C, antifungal activity was evaluated in a quantitative spray co-exposure apparatus as shown in the lower panel of Fig. 1C. Under the five-second co-spray condition established for this study, approximately 25,000 particles within the 400–1000 nm range corresponded to a peak concentration of 67.6 particles/cm^3^ in the 370 cm^3^ sealed reaction vessel within a short exposure period. Importantly, the delivered particle population was still dominated by the 400–500 nm fraction, consistent with the size-distribution profile observed during nanosalt preparation.

**Table 2:** Particle size and sedimentation parameters of nanosalt particles

Particle size (nm)	Time/descending distance (min/m)	Subsidence acceleration (μm/s^2^)
400–500	**707.14**	**0.00206**
500–750	**857.14**	**0.00108**
750–1000	**621.43**	**0.00159**

### Fungicidal activities of nanosalts against conidia from different *Aspergillus fumigatus* isolates

**Fig. 2:**
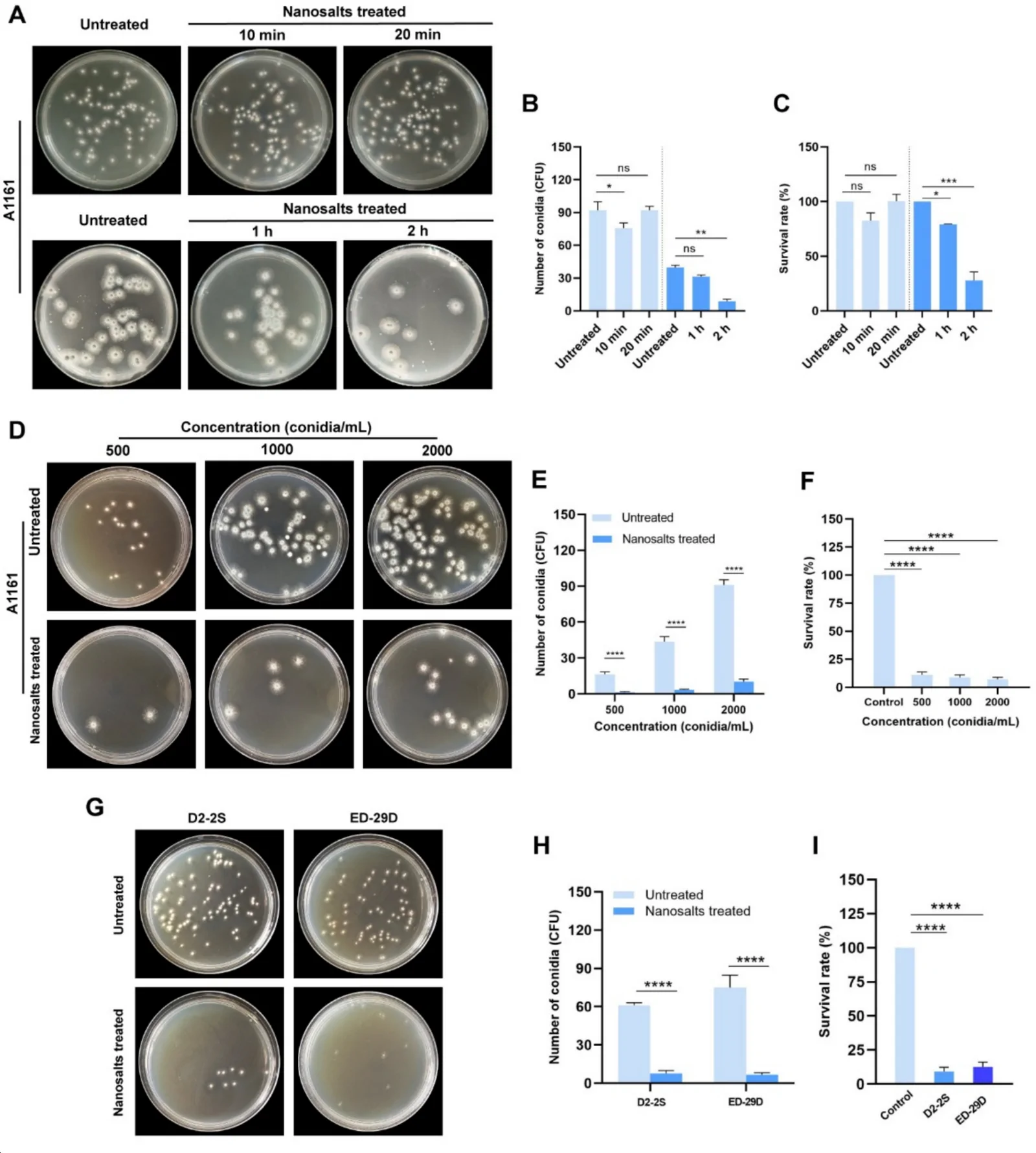
Nanosalt exhibits potent activity against both azole-susceptible and azole-resistant *Aspergillus fumigatus* conidia. **A**–**C** Time-dependent survival of azole-susceptible *A. fumigatus* A1161 conidia after nanosalt treatment. Representative CFU plates (**A**), CFU quantification (**B**), and survival rates (**C**) are shown. **D**–**F** Fungicidal activity of nanosalt against azole-susceptible *A. fumigatus* A1161 conidia at different initial inoculum concentrations. Representative CFU plates (**D**), CFU quantification (**E**), and survival rates (**F**) are shown. **G–I** Fungicidal activity of nanosalt against the environmental azole-susceptible isolate D2-2S and azole-resistant isolate ED-29D. Representative CFU plates (**G**), CFU quantification (**H**), and survival rates (**I**) are shown. Data are presented as the mean ± SD from three independent experiments; ns, not significant, *P < 0.05, **P < 0.01, ***P < 0.001, ****P < 0.0001

To investigate the antifungal efficacy of nanosalts against *A. fumigatus*, the azole-susceptible clinical reference strain A1161 was selected as the main strain. To exclude conidial mortality caused simply by mechanical collision during simultaneous spraying, viable cells were harvested at multiple time points for CFU quantification. As presented in Fig. 2A–C, conidia maintained high viability, with no significant difference in survival rate relative to the untreated control when the contact time between conidia and nanosalt was shorter than 20 min and quantification was performed immediately. However, when the contact time between conidia and nanosalt was 2 h, viable conidia counts declined significantly versus the control, indicating that nanosalt-mediated fungicidal activity requires sufficient contact time after co-spraying. Subsequent CFU assays were therefore evaluated at the 2 h contact time between conidia and nanosalt in a quantitative spray co-exposure apparatus. Conidia at three concentrations (500, 1000, and 2000 conidia/mL) were nebulized, and then collected, washed with PBS, plated, and quantified by CFU counting. nanosalt-treated groups showed drastically reduced CFU counts (Fig. 2D, E), with survival rates of merely 11.0%, 8.8%, and 7.2%, respectively (Fig. 2F). To further assess whether the antifungal activity was retained against additional environmental isolates, one azole-susceptible isolate and one azole-resistant isolate were tested under the same conditions. As shown in Fig. 2G–I, nanosalts exerted potent antifungal effects on both isolates. Collectively, these findings demonstrate that nanosalts have efficient fungicidal activity against *A. fumigatus* conidia under the defined chamber conditions used here.

### Nanosalts have comparable fungicidal activities against *Aspergillus flavus* and *Aspergillus niger*

**Fig. 3:**
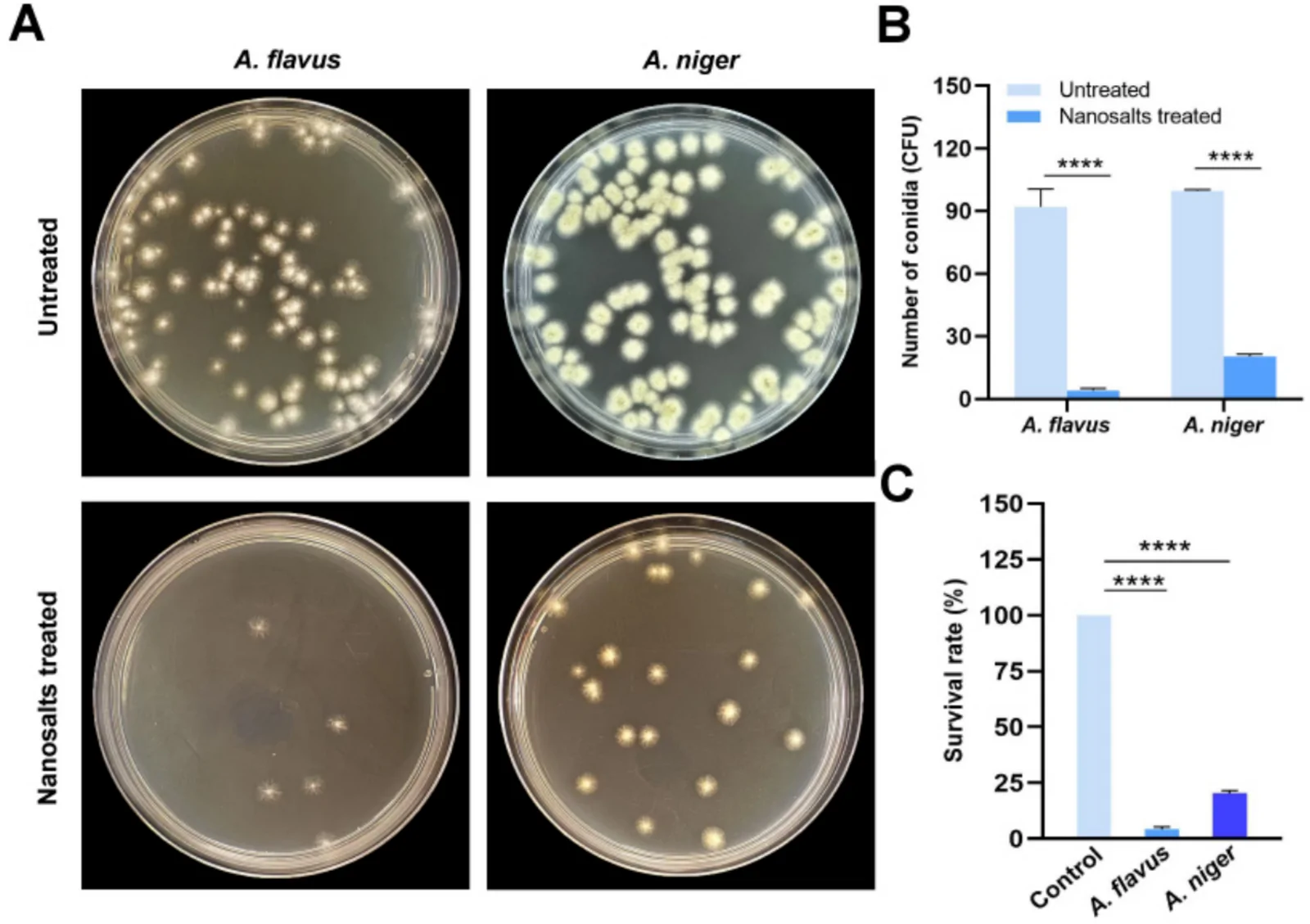
Nanosalt exhibits antifungal activity, demonstrating potent efficacy against *Aspergillus flavus* and *Aspergillus niger*. Representative CFU plates (**A**), CFU quantification (**B**), and survival rates (**C**) of *A. flavus* and *A. niger* conidia following nanosalt treatment are shown. Statistical significance was analyzed as described in Methods; ****P < 0.0001

Beyond *A. fumigatus*, *A. flavus* and *A. niger* are also clinically important species and were included as additional filamentous fungal models relevant to environmental exposure (Casalini et al. 2024). Using the same chamber protocol established for *A. fumigatus*, conidial suspensions adjusted to 2,000 conidia/mL were co-exposed to nanosalts for 5 s in a sealed container. As displayed in Fig. 3A and B, nanosalt treatment drastically reduced their CFU counts. The survival rates were 4.3% for *A. flavus* and 20.4% for *A. niger* (Fig. 3C), with consistent results across replicates. These findings indicate that nanosalts exert marked in vitro antifungal activity against multiple *Aspergillus* species under the tested conditions.

### Nanosalts also have fungicidal effects against medically important opportunistic yeasts C*andida albicans *and *Cryptococcus neoformans*

To determine whether nanosalt activity extended beyond the airborne *Aspergillus* model, we additionally tested two medically important yeasts for human opportunistic infection, *C. albicans* and *C. neoformans*. These experiments were designed to assess the breadth of in vitro antifungal activity rather than to inhibit routine hospital airborne transmission, particularly in the case of *C. albica*ns which is the most common cause of opportunistic yeast infections but not a typical airborne pathogen (Lee et al. 2021; Lopes and Lionakis 2022). *C. neoformans* is relevant to inhalational acquisition from environmental sources, but its ecology differs substantially from that of *Aspergillus* in healthcare settings (Iyer et al. 2021; Zhao et al. 2023).

**Fig. 4:**
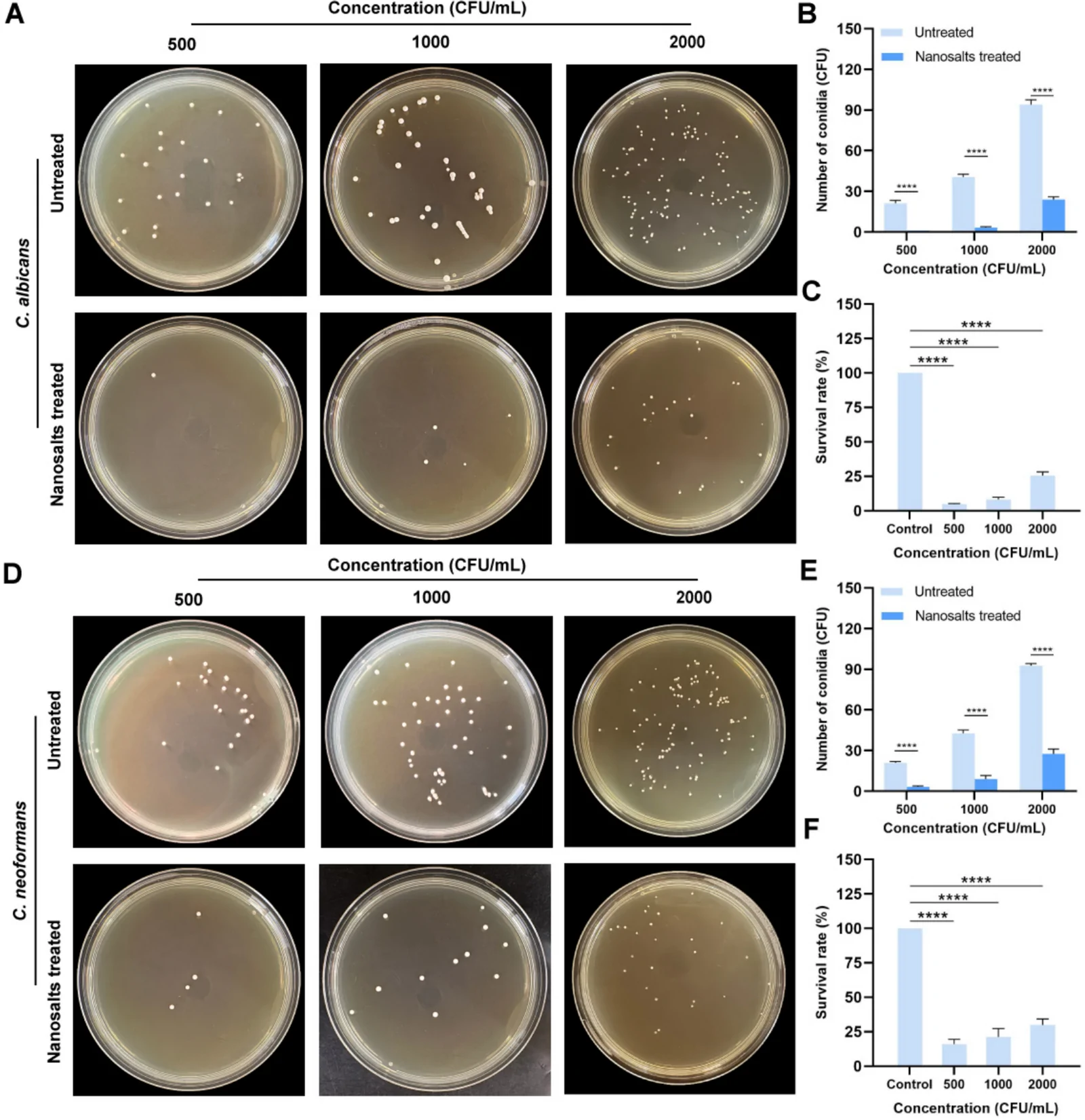
Nanosalt demonstrates fungicidal effects against the opportunistic yeasts *Candida albicans* and *Cryptococcus neoformans*. **A**–**C** Representative CFU images, CFU quantification, and survival rates of *C. albicans* following nanosalt treatment. **D**–**F** The identical procedure was employed to assess the data on the fungicidal efficacy of nanosalts against *C. neoformans*. Error bars indicate the SD from three independent experiments; ****P < 0.0001

We further evaluated the antifungal efficacy of the as-prepared nanosalts against these two pathogens. Nanosalts exhibited potent fungicidal activity. As shown in Fig. 4A and D, CFU was markedly reduced in nanosalt-treated groups across all tested inoculum concentrations (500, 1000, 2000 CFU/mL) compared with untreated controls. Quantitative analysis confirmed significantly lower CFU counts in treatment groups (Fig. 4B and E). At the lowest concentration of 500 CFU/mL, survival rates were merely 4.0% for *C. albicans* and 13.3% for *C. neoformans* (Fig. 4C and F). The significant fungicidal effect was shown across all concentrations, demonstrating that nanosalt also has broad in vitro activity against these yeast pathogens.

### Nanosalt-mediated time-dependent elevation of reactive oxygen species (ROS) in *A. fumigatus*

**Fig. 5:**
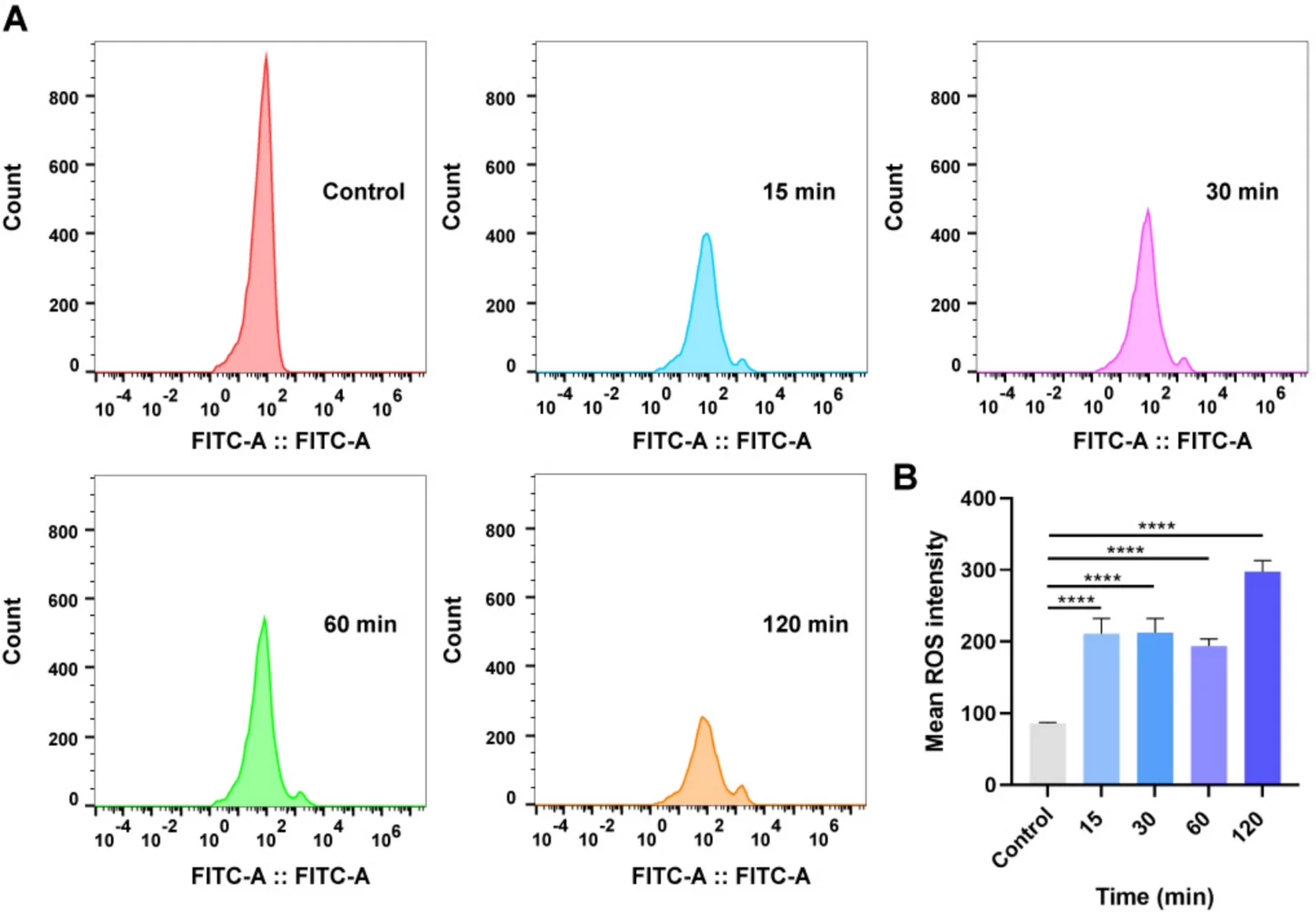
Nanosalt mediates the time-dependent increase in ROS in *Aspergillus fumigatus* conidia. **A** Histogram of ROS fluorescence distribution in nanosalt-treated *A. fumigatus* conidia under different treatment durations. **B** Quantification of mean ROS fluorescence intensity in nanosalt-treated *A. fumigatus* conidia under different treatment durations relative to (**A**), ****P < 0.0001

Given the broad-spectrum antifungal activity of nanosalts, *A. fumigatus* A1161 was employed as the model organism for possible mechanistic experiments. Intracellular ROS levels were detected following the commercial test kit protocol by flow cytometry after DCFH-DA labeling at 15, 30, 60, and 120 min of nanosalt-conidia co-incubation. As illustrated in Fig. 5A, flow cytometry fluorescence histograms demonstrated a time-dependent shift of the fluorescence peak to higher intensity with prolonged nanosalt-conidia co-incubation, accompanied by a prominent reduction in the cell count corresponding to the peak. The controls had a low-intensity fluorescence peak with maximum cell counts. No significant discrepancies in peak shift magnitude or cell count were observed between the 15 and 30 min treatment groups. The 60 min group exhibited further elevated fluorescence intensity and noticeably decreased cell counts. The 120 min group presented the highest fluorescence intensity, with intact cell counts significantly lower than those of all other treatment groups and the control. Figure 5B quantifies the mean ROS fluorescence intensity. Statistical analysis revealed extremely significant differences among all groups (P < 0.001). The control group had the lowest intensity (85.8 ± 1.26), while the 120 min group reached the peak value of 297.7 ± 15.56, a 2.48-fold increase relative to the control. These data indicate that nanosalts treatment is associated with a time-dependent elevation of intracellular ROS in fungal conidia.

### Nanosalts are capable of disrupting conidial cell wall and membrane integrity

**Fig. 6:**
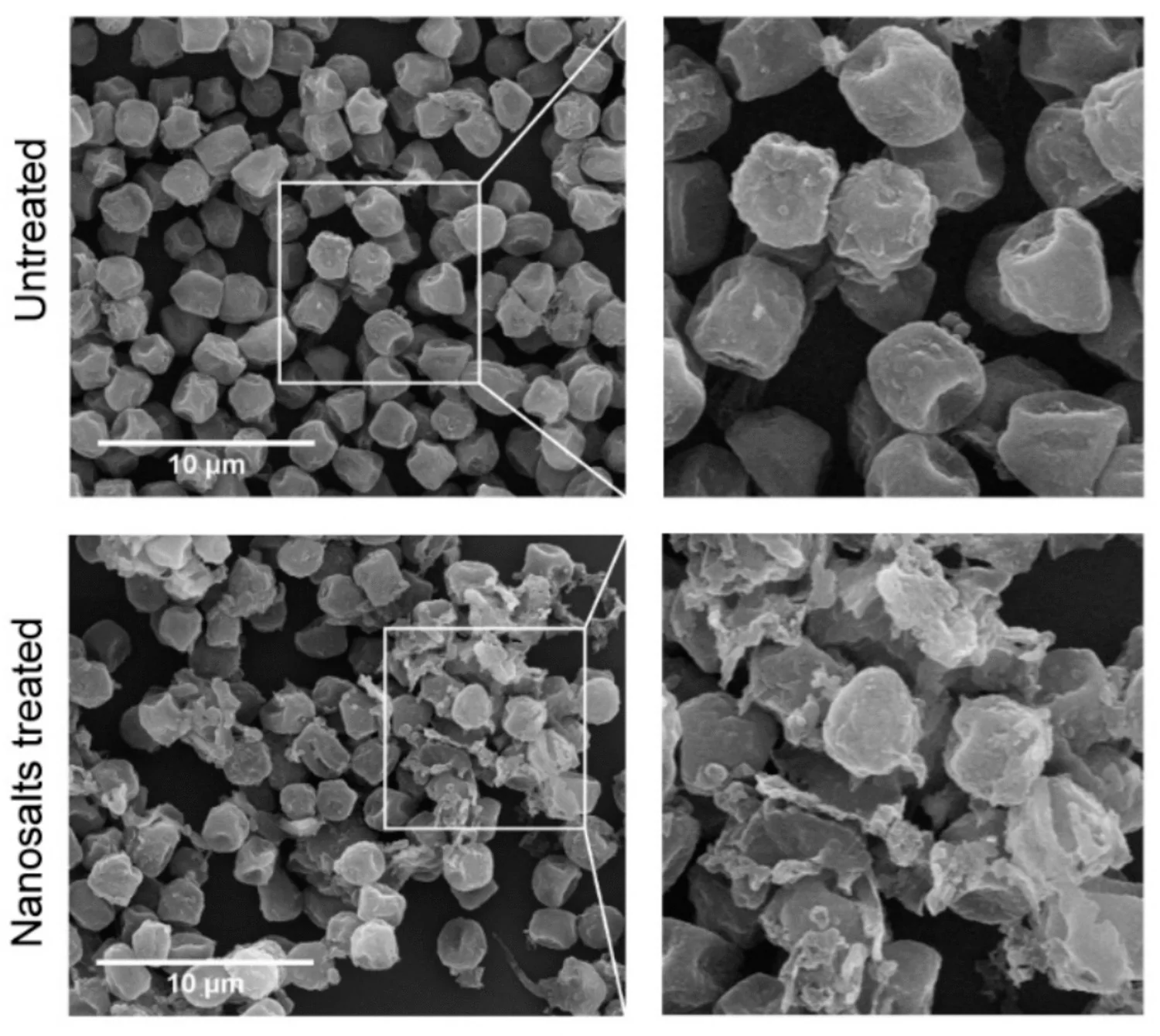
Nanosalt treatment induces fragmentation and structural disruption in *Aspergillus fumigatus* conidia. Examination of nanosalt-induced structural changes in *A. fumigatus* conidia by SEM. Scale bar = 10 μm

The preceding ROS detection results indicated that *A. fumigatus* conidia underwent oxidative damage alongside structural disruption. To intuitively validate the detrimental effects of nanosalts on conidial morphology and surface structure, scanning electron microscopy (SEM) was utilized to observe micromorphological alterations in conidia after various exposure durations. Fresh *A. fumigatus* A1161 conidia were harvested from YAG medium plates, filtered, counted, and prepared into a conidial suspension. As presented in Fig. 6, untreated conidia appeared plump and spherical or subspherical, with intact structures and smooth surface features. Conversely, nanosalt-treated conidia displayed severe structural damage, presenting irregular morphology with substantial fragmentation.

**Fig. 7:**
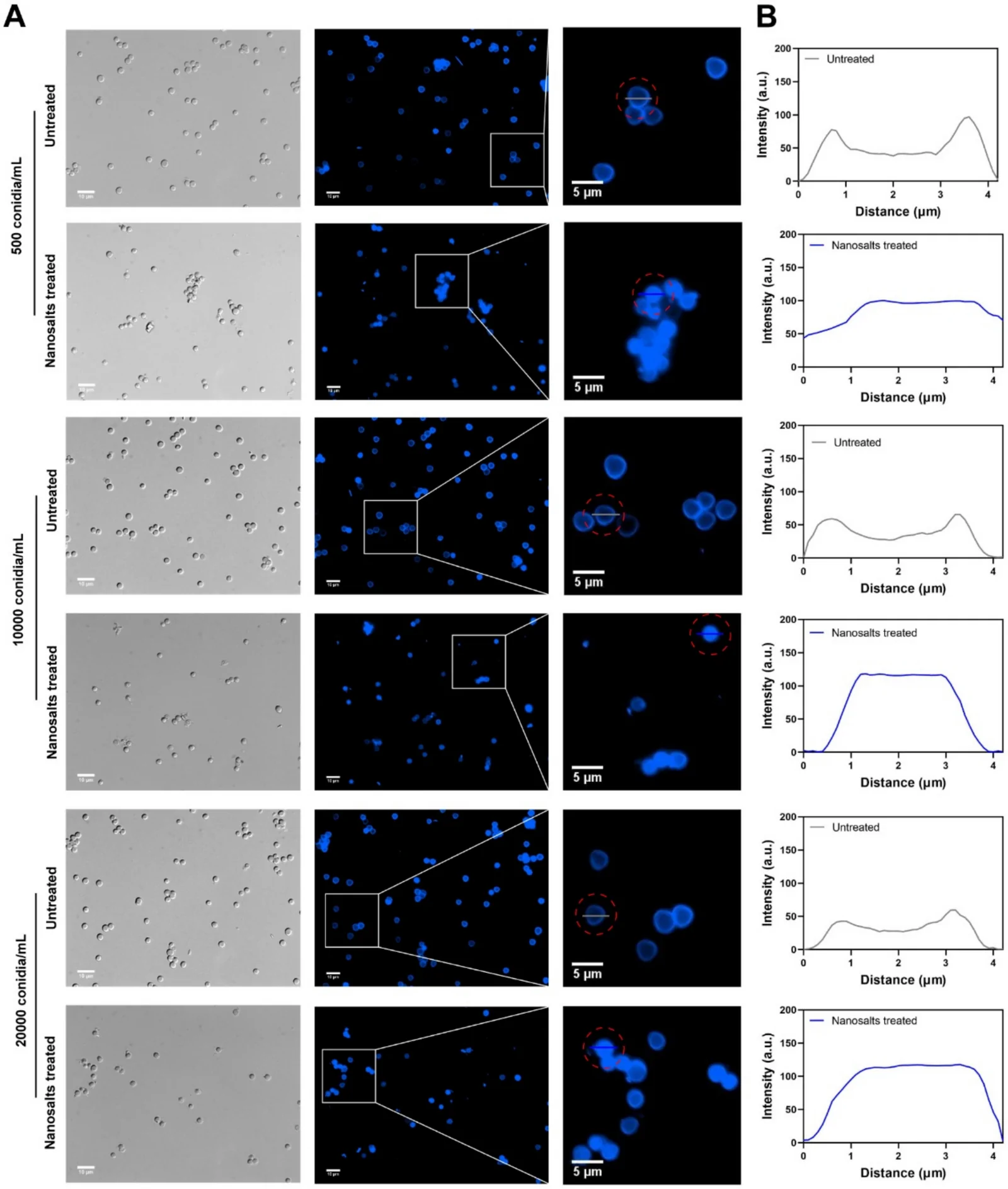
CFW staining demonstrates nanosalt-induced cell wall integrity impairment in *Aspergillus fumigatus* conidia. **A** Assessing cell wall changes and conidia fluorescence intensity by CFW staining in *A. fumigatus* conidia after treatment with different nanosalt concentrations. **B** Fluorescence intensity profiles of representative conidia selected from the stained images in panel (**A**) were analyzed using ImageJ. Scale bars = 10 μm and 5 μm

As SEM only reveals surface morphological changes, the impact on cell wall composition remained undetermined. Thus, Calcofluor White (CFW), a chitin-specific fluorescent dye, was applied to assess cell wall variations across treatment groups (Wang et al. 2021). CFW preferentially binds to β−1,4 and β−1,3 polysaccharide chains, emitting intense blue fluorescence upon excitation, making it ideal for labeling fungal cell walls. As shown in Fig. 7A, untreated conidia exhibited uniform morphology with concentrated, well-defined fluorescent signals. In contrast, treated conidia showed aggregation, deformation and diffuse fluorescence, indicating compromised cell wall integrity. The fluorescence intensity quantification profiles in Fig. 7B further demonstrated that sharp distinct peaks in the control group corresponded to cell wall of conidia, while broad, flattened peaks in the treated group indicated damaged, fused structures of cells.

**Fig. 8:**
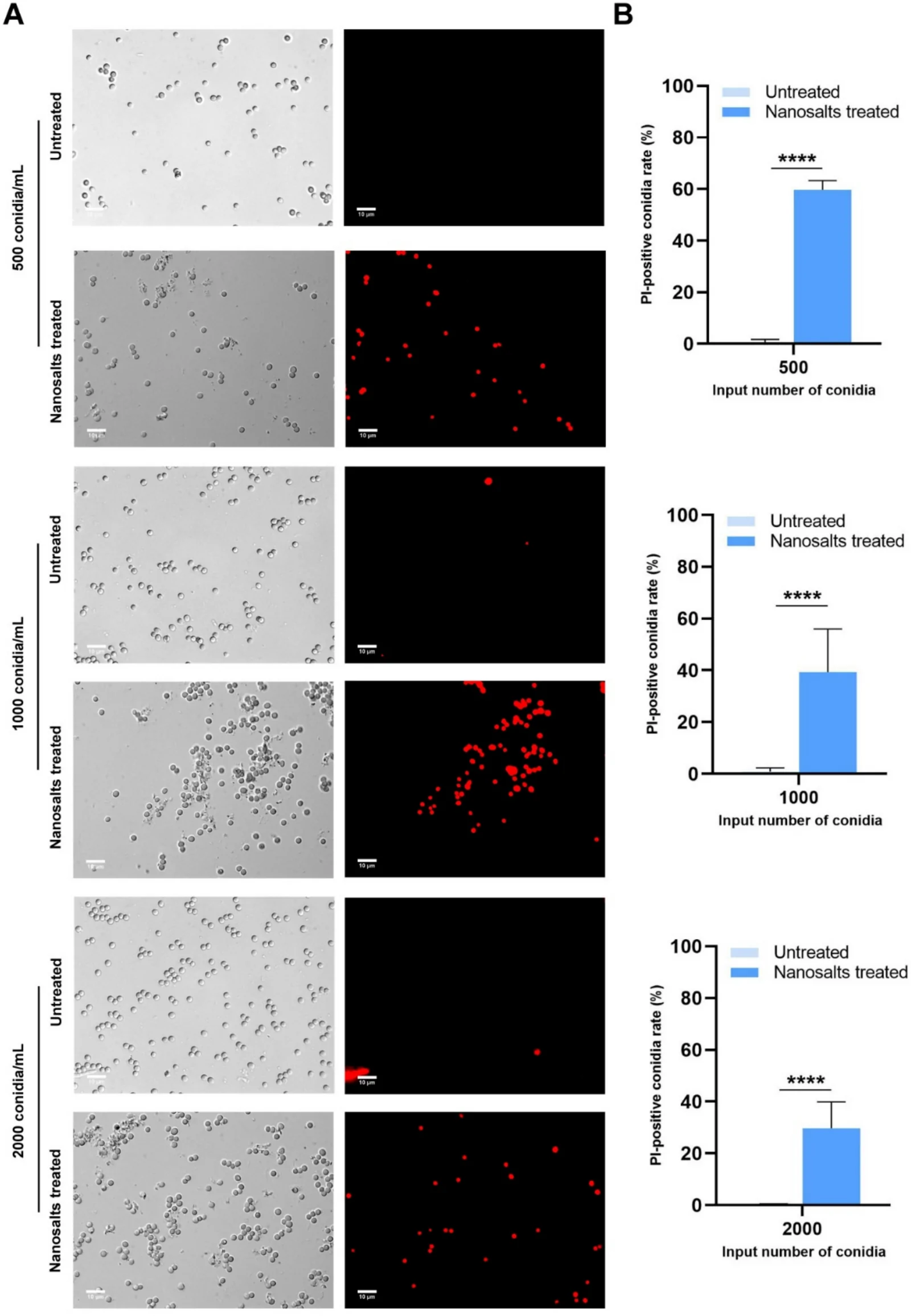
Loss of cell membrane integrity was observed by PI staining in *Aspergillus fumigatus* conidia with nanosalt treatment. **A** The viability of *A. fumigatus* conidia following nanosalt treatment was directly assessed by PI staining. **B** The corresponding percentage of PI-positive cells was quantified relative to (**A**). Scale bar = 10 μm. Statistical significance was analyzed as described in Methods; ****P < 0.0001

Propidium iodide (PI) staining was subsequently performed to evaluate conidial viability (Shailaja et al. 2022). PI cannot permeate intact live cell membranes and only stains dead cells by binding to DNA. Figure 8A shows no fluorescence in untreated conidia, but strong red fluorescence in nanosalt-treated groups, indicating extensive conidial death. Quantitative analysis (Fig. 8B) confirmed a drastically elevated PI-positive rate in treated groups, with extremely significant differences (****, *P* < 0.0001) at all tested conidial concentrations. While the PI-positive rate of cells was declined slightly with increasing initial cell density, the absolute number of dead conidia still increased, suggesting a concentration-dependent antifungal effect. Collectively, these findings demonstrate that nanosalts kill conidial cells of *A. fumigatus* by disrupting cell wall and cell membrane integrity.

## Discussion

The present study demonstrates that nanosalt particles synthesized by anti-solvent precipitation and high-energy nanonization possess broad in vitro fungicidal activity against several medically important fungi under a designed spray co-exposure assay. Among the organisms tested, *Aspergillus* spp., especially *A. fumigatus*, are the most directly relevant to the concept of airborne environmental decontamination because their conidia are readily aerosolized and are recognized contaminants in healthcare environments. By contrast, the experiments with *C. albicans* and *C. neoformans* should be interpreted primarily as evidence of a broader antifungal spectrum. Probably, these yeast results suggest that nanosalt may also merit future evaluation for spray disinfection of contaminated surfaces, wall materials, and other contact-relevant settings.

**Fig. 9:**
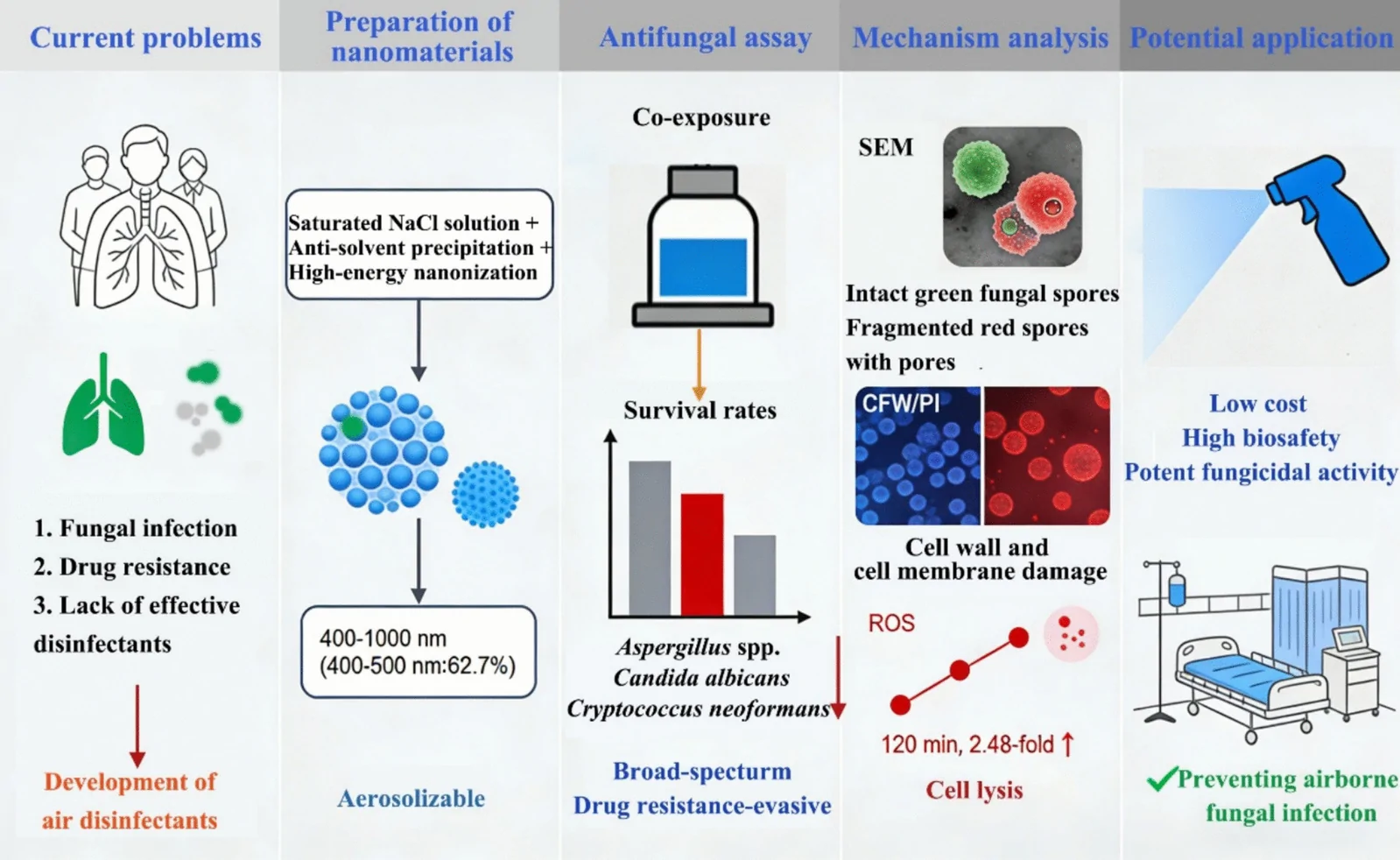
Proposed model summarizing the in vitro antifungal activity of atomized nanosalt particles and the associated cellular damage observed in this study

Our data provide preliminary mechanistic evidence that nanosalt exposure is associated with severe fungal cell-envelope injury and ROS accumulation. SEM revealed fragmentation and surface disruption, CFW staining supported impairment of cell wall integrity, and PI staining demonstrated loss of membrane integrity and cell death. A time-dependent increase in intracellular ROS was also observed in *A. fumigatus* conidia. Because sodium chloride is not a redox-active nanomaterial, we do not interpret this finding as direct ROS generation by nanosalt itself. Rather, the most plausible interpretation is that ROS accumulation represents a secondary cellular stress response triggered by nanosalt-induced osmotic imbalance and structural damage to the cell wall and membrane. Collectively, based on the observed findings, the antifungal activity of nanosalt in this study can be interpreted as a consequence of cell membrane and cell wall damage, accompanied by the secondary accumulation of ROS. These observations are summarized in the proposed model shown in Fig. 9.

The differential susceptibility observed across fungal species likely reflects differences in cell-envelope architecture, aggregation behavior, and stress-buffering capacity. Importantly, nanosalt also retained activity against both azole-susceptible and azole-resistant *A. fumigatus* isolates, supporting the view that its fungicidal action is independent of classical azole-resistance pathways and is instead dominated by physical and physicochemical injury under the present assay conditions (Sette et al. 2013; Sousa et al. 2017).

Meanwhile, the current study still has important limitations, particularly regarding biosafety. The co-spray chamber used here is a simplified proof-of-concept exposure system and does not fully reproduce real hospital airflows, humidity, travel distance, ventilation, particle deposition dynamics, or human occupancy. Therefore, the present results should not be overinterpreted as direct evidence that nanosalt is already effective for clinical air-disinfection. Regarding biosafety, a previous mouse bone marrow micronucleus assay of NaCl extrafine powder provided preliminary evidence of no obvious genotoxicity under the tested conditions (Chen et al. 2013). However, because the intended application involves aerosolized or dry-powder exposure, this preliminary evidence cannot replace dedicated respiratory-safety evaluations, including inhalation toxicity, pulmonary inflammation, lung-microbiome effects, and environmental-safety assessments. Thus, the present data support nanosalt only as a promising candidate dry antifungal material that merits further translational evaluation, rather than as a material ready for practical clinical or environmental air-disinfection use (Liao et al. 2025).

Future work should include larger and better-controlled aerosol chambers, airflow and humidity simulations, time-of-flight and deposition analyses, comparison with benchmark environmental disinfectants, dedicated respiratory and environmental biosafety studies, including inhalation toxicology, dose-dependent pulmonary responses, particle retention and clearance, environmental fate, and ecotoxicological assessment (Aranke et al. 2021). For the non-airborne yeast findings, future studies should test practical spray-disinfection performance on contaminated surfaces, wall materials, and other fomites, where broad antifungal surface decontamination may be the more relevant application scenario.

## Data Availability

All data supporting the findings of this study are available within the paper.
